# Transient small bowel intussusception in an adult: case report with intraoperative video and literature review

**DOI:** 10.1186/s12893-015-0020-6

**Published:** 2015-04-03

**Authors:** Hager Aref, Abrar Nawawi, Abdulmalik Altaf, Murad Aljiffry

**Affiliations:** Department of Surgery, Faculty of Medicine, King Abdulaziz University, Jeddah, Saudi Arabia

**Keywords:** Intussusception, Adult intussusception, Transient intussusception

## Abstract

**Background:**

The term intussusception refers to invagination of a segment of the gastrointestinal tract into the lumen of an adjacent segment. This is a rare entity and it is more prevalent in children and less common in adults. The diagnosis of intussusception in adults is difficult as a result of the nonspecific signs and symptoms. As there are many common causes of acute abdomen, intussusception should be considered when more frequent etiologies have been ruled out. The laparoscopic approach offers both a diagnostic option and a therapeutic one for intussusception in adults.

**Case presentation:**

We report a forty-one year old male patient, who presented to our Emergency Department complaining of peri-umbilical pain associated with nausea and vomiting for 1 day. Diagnosed with transient small bowel intussusception without any obvious underlying pathology. This report is the first to present an intra-operative video showing the small bowel intussuscepting and reducing spontaneously. Furthermore, the authors present a review about this rare condition, including previously reported similar cases in literature.

**Conclusion:**

Transient intussusception is extremely rare and is a challenging condition. Imaging techniques, especially CT scan, are helpful in the diagnosis of intussusception. However, laparoscopy offers the advantage of distinguishing transient intussusception from persistent intussusception.

**Electronic supplementary material:**

The online version of this article (doi:10.1186/s12893-015-0020-6) contains supplementary material, which is available to authorized users.

## Background

Intussusception has long been discussed in medical literature. Barbette of Amsterdam described the first case in 1674 [1]. In 1742, Cornelius Henrik Velse performed the first successful operation on adult intussusception [2].

Intussusception is a rare form of bowel obstruction in adults, which is defined as the telescoping of a proximal segment of the gastrointestinal tract, into the lumen of the adjacent distal segment [3]. The overall incidence of intussusception in adults is around 2–3 cases per 1,000,000 of the general population annually [4]. Intussusception in adults is usually secondary to an existing pathology; in pediatric population, however it is mostly primary in origin. Furthermore, intussusception in adulthood differs from that in childhood in presentation, diagnosis and treatment. In adults, 90% of intussusceptions occur in small or large bowel and 10% affects the stomach or surgically created stomas [5]. While 60% of colonic intussusceptions in adults are induced by malignant tumor as the lead point, 30% of small bowel intussusceptions are caused by malignancy [6,7].

About 10% of small bowel intussusceptions in adults are idiopathic [8]. This article describes a case of a male adult with idiopathic transient small bowel intussusception.

## Case presentation

We present the case of a forty-one year old male patient with previous medical history of Diabetes Mellitus, who never had any surgical intervention. He presented to our Emergency Department complaining of peri-umbilical pain for 1 day. The pain was moderate in severity and colicky in nature, associated with nausea and vomiting. There was no history of hematemesis, melena, or hematochezia. His vital signs were unremarkable. Abdominal examination revealed moderate tenderness in the peri-umbilical region with no rebound tenderness or guarding and no organomegaly. Bowel sounds were audible, and rectal examination was normal. His laboratory investigations were within normal range.

**Figure 1:**
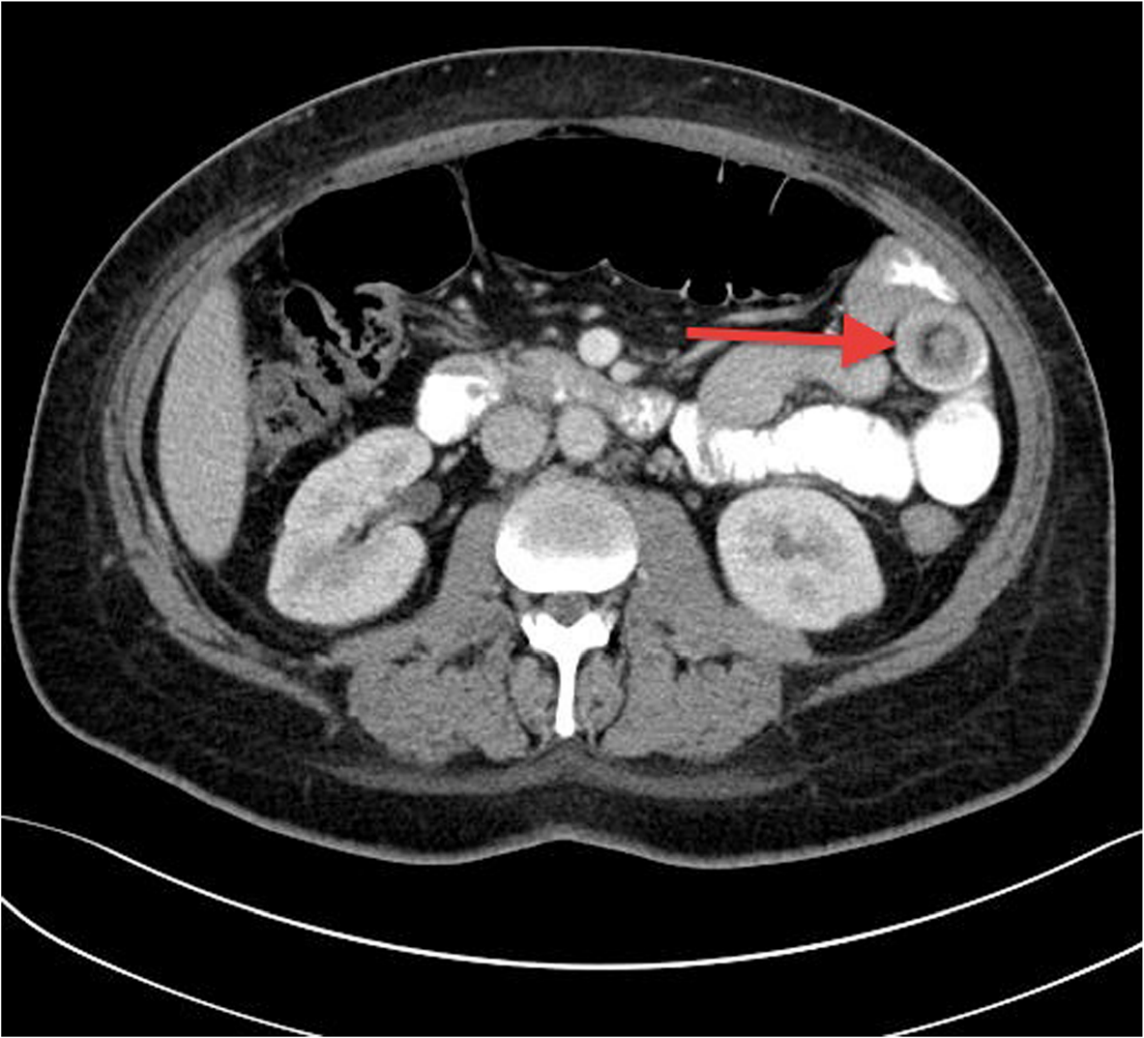
**A CT scan of the abdomen (transverse section); showing small bowel loops filled with oral contrast and a target sign appearance at the left side of the abdomen (arrow).**

**Figure 2:**
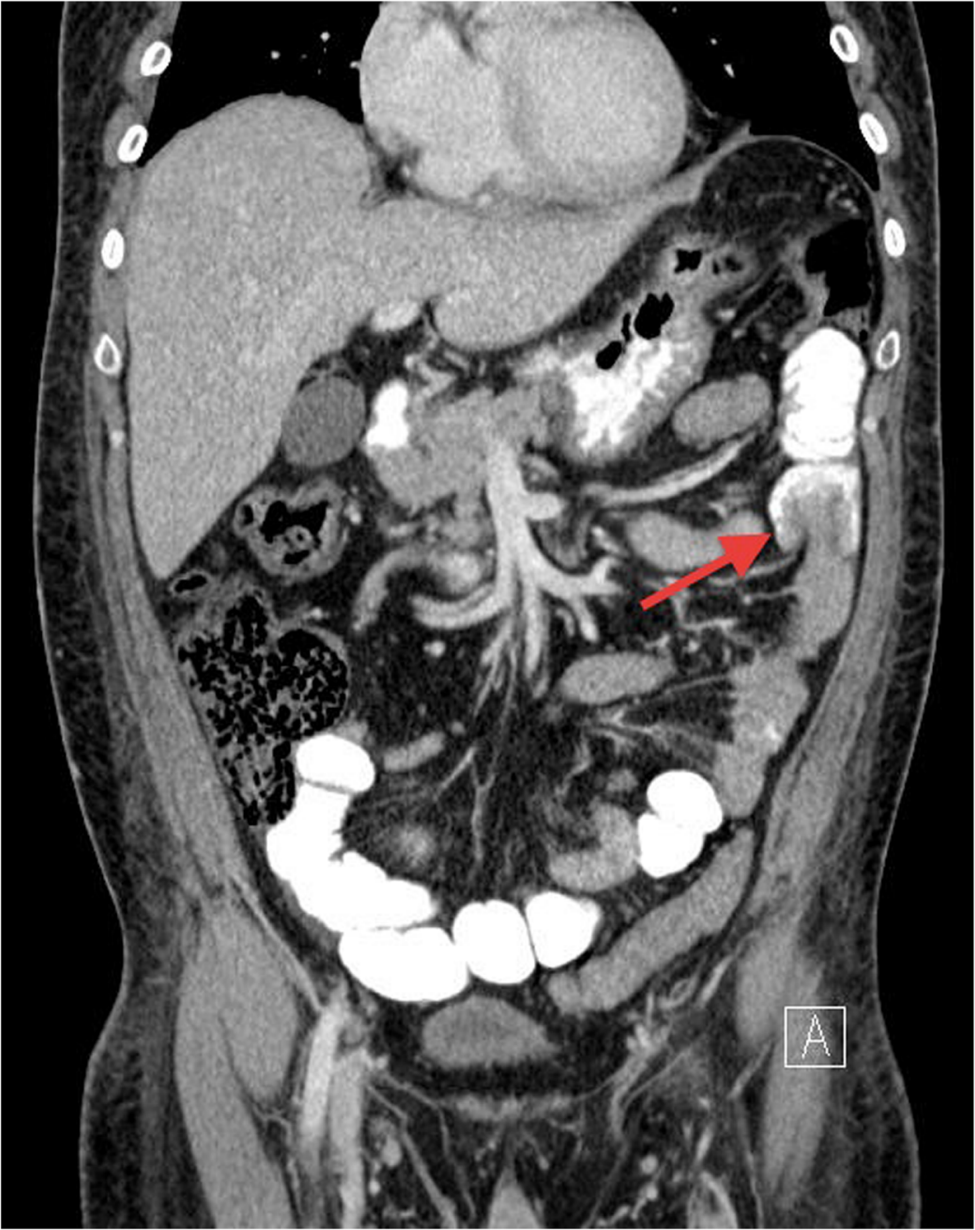
**Abdominal CT scan (coronal section); showing a segment of small bowel loop.** Invaginated into a proximal bowel loop, indicating small bowel intussusception (arrow). There were no signs of bowel ischemia nor obstruction.

**Figure 3:**
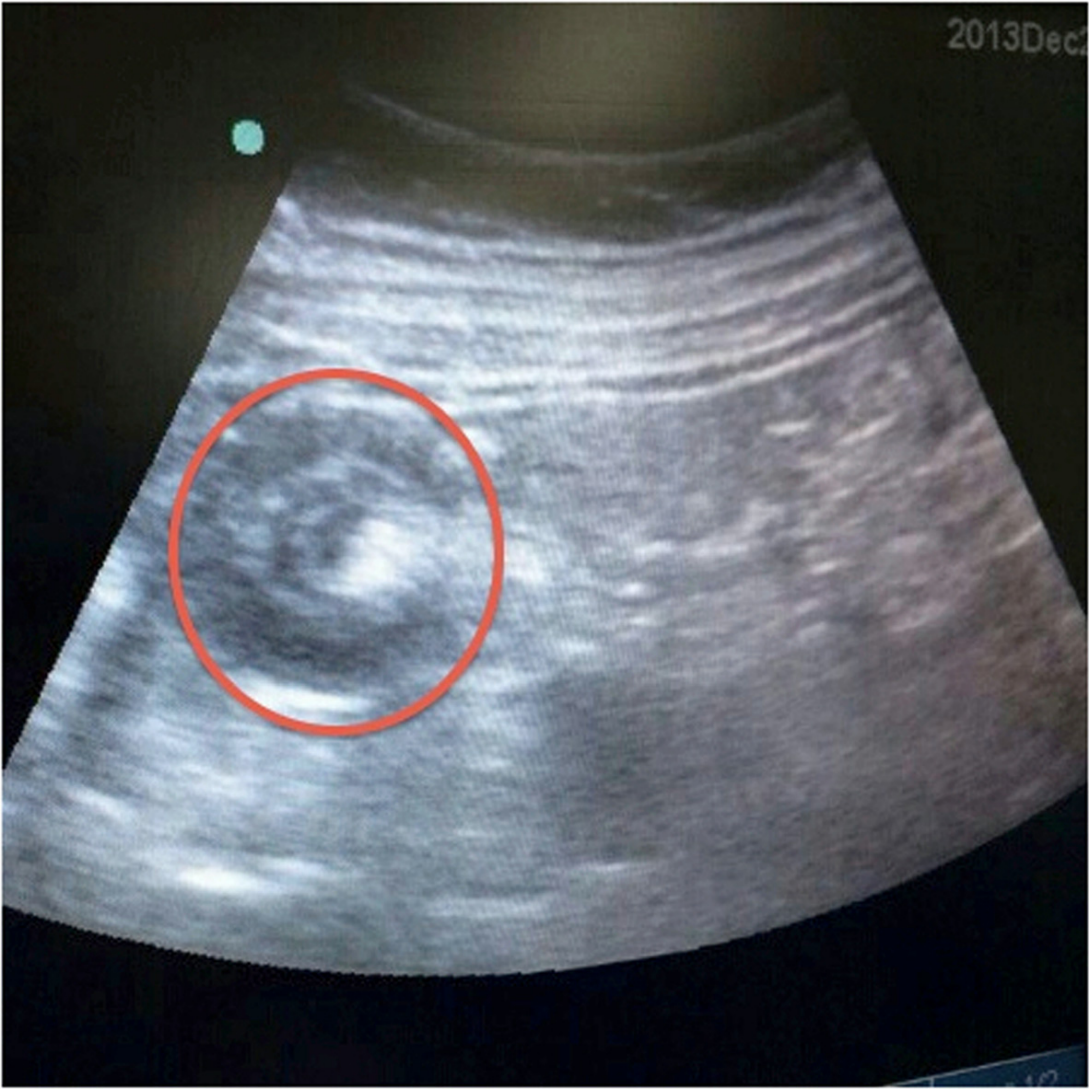
**An intraoperative ultrasound, showing the classic target sign appearance of intussusception.**

He underwent Computed Tomography (CT) of the abdomen and pelvis, which revealed a short segment of small bowel intussusception at the left upper quadrant with a target sign appearance. There were no signs of bowel obstruction or ischemia. (Figures 1 and 2). Since the patient’s symptoms of pain and vomiting were persistent and the CT was showing small bowel intussusception in a diabetic patient who has no known risk of developing transient intussusception, in addition to the surgeons concern of irreducibility and possible presence of underlying pathology, the decision was made to take him for laparoscopic exploration. In the operating room following general anesthesia, the patient was placed in supine position with split legs. Transabdominal ultrasound was performed prior to skin cut and it showed the small bowel intussusception with target sign (Figure 3) confirming the CT finding. The exploration was performed using three ports. On inspecting the peritoneum, there was no free fluid nor were there signs of inflammation. The small bowel was inspected from the ligament of Trietz till the ileo-cecal valve. Multiple segments of the small bowel were observed intussuscepting and reducing spontaneously (Additional file 1). During the running of the small bowel no visible masses or other pathology were identified.

Following that, we made a small laparotomy of 3cm in size by enlarging the 12mm peri-umbilical port. This was used to manually examine the bowel for small nonvisualized intra-luminal or intra-mural masses or polyps; the result was also negative. The patient had uneventful recovery and discharged home on day 3 post operation. The patient was informed and educated about his condition, was prescribed anti-spasmodic for symptomatic relief when needed and was instructed to return to emergency care if an attack does not resolve. The patient also had a small bowel follow-through study 4 weeks post surgery, which was normal. There were no adverse or unanticipated events.

All data reported in the manuscript have been visualized and then approved by our University Hospital Ethics Committee and all procedures carried out on the patients were in compliance with the Helsinki Declaration. Furthermore, the authors confirm that a written informed consent was obtained from the patient for publication of this case report and accompanying images.

## Discussion

Intussusception is defined as the invagination of a proximal segment of the gastrointestinal tract, into the lumen of the contiguous distal segment [3]. Intussusception can occur at any age but it is most common in children. About 5% of cases of intussusception are present in adults and causes 1%-5% of intestinal obstructions in this population [9]. The prevalence is equal between adult genders. Adult intussusception is usually caused by a pathologic lead point within the bowel and over half of the cases were triggered by a malignant lesion [10,11].

The mechanism behind intussusception could be explained by the presence of a bowel lesion that alters the normal peristaltic movements and serves as a lead point for intussusception [11]. On the other hand, the mechanism of the rare entity of transient small bowel intussusception, as in our case, is not well described in medical literature. Transient intermittent intussusceptions were reported in literature in patients with celiac [3] or Crohn’s [12] disease. However, they are frequently idiopathic and reduce spontaneously without any surgical intervention. They may become symptomatic when spontaneous reduction is unsuccessful. The transient intermittent small bowel intussucesption is a rare condition with only few similar cases reported in literature (Table 1). Catalano reported 5 cases of transient small bowel intussusception. He described intussusception as momentary dysrhythmic contractions resulting in abnormal peristalsis. As a short loop of the small bowel contracts abnormally, it forces intestinal wall inward, creating the intussusceptum [13]. Napora et. al reported 2 cases of transient, idiopathic adult jejunal intussusception. Both patients complained of nonspecific abdominal pain and nausea and were diagnosed with intussusception by abdominal CT scan. In both cases, no underlying bowel abnormality was identified and neither required a bowel resection, which resembles our case report [14].

**Table 1:** **Literature review of transient intussusception in adults**

**Title**	**Author**	**Year**	**Age**	**Gender**	**Presentation**	**Treatment**	**Pathology**
***Transient small bowel intussusception: CT findings in adults***	Catalano, et al. [13]	1997	56	M	No complain, intussusception was an incidental finding on CT scan	Conservative with Follow up	no underlying bowel abnormality
			60	F			
			65	M			
			18	M	Night sweats, fever, weight loss		
			44	M	No complain, intussusception was an incidental finding on CT scan		
***Transient adult jejunal intussusception***	Napora, et al. [14]	2002	19	F	nonspecific abdominal pain and nausea	exploratory laparotomy	no underlying bowel abnormality
			39	M	abdominal pain and nausea	Conservative	
***Idiopathic intussusception in male adult: A case report***	Makrodimou et al. [20]	2002	43	M	colicky epigastric pain	Diagnostic laparoscopy	No underlying pathology
***Jejunal Intussusception as an Unusual Cause of Abdominal Pain in an Adult***	Mehta, et al. [21]	2009	29	M	peri-umbilical abdominal pain	Conservative	no underlying bowel abnormality
***Adult intussusception - 14 case reports and their outcomes***	Guillén, et al. [22]	2010	58	F	Abdominal pain	Conservative	No underlying pathology
			54	F			Pancolitis
			44	M			Nodular lymphoid hyperplasia
			17	M			No underlying pathology
***This Isn’t Child’s Play: Transient Intussusception In Adults***	Bennani^,^ et al. [23]	2013	22	M	intractable nausea, vomiting, and abdominal pain	Conservative	no underlying bowel abnormality
***Transient intussusceptionz rare cause of abdominal pain in cystic fibrosis***	Artul, et al.	2013	26	M	abdominal pain, nausea and vomiting.	Conservative	Cystic fibrosis

In adults, intussusception is usually accompanied with intermittent abdominal pain, nausea, vomiting, constipation, melena, weight loss, and fever. Abdominal pain is considered to be the most common symptom, presenting in 70-100% of cases [15]. Transient small bowel intussusception can carry a further challenge as it often present with nonspecific symptoms and signs [14]. It has been reported that transient small bowel intussusception can be completely asymptomatic [13].

There are multiple radiographic tools that can aid the surgeon in the diagnosis of intussusception, These include plain abdominal films, ultrasound and CT scans of the abdomen. A plain abdominal film may show the typical features of distal small bowel obstruction. Ultrasound may show a Bull’s eye sign of the involved segment of bowel [16]. However, both abdominal x-rays and ultrasound imaging are of limited diagnostic value in adults. The diagnosis of this condition is often made by using abdominal CT scan. It is the most useful diagnostic tool with a diagnostic yield of around 78%, and it also helps in identifying the underlying cause [17]. A “target sign” may be seen on the sagittal view of the abdominal CT. The distended loop of bowel appears thickened, representing two layers of bowel [18]. In addition, abdominal CT can be helpful in identifying the lead point in intussusceptions, if present. In cases where the diagnosis of intussusception is uncertain despite CT imaging, utilizing laparoscopic exploration for the diagnosis of adult intussusception is considered safe and doable. Furthermore, it can be used safely to manage intussusception, as in the described case [19].

Treatment is almost always surgical in adults with pathological intussusception, where resection and primary anastomosis of the involved segment of bowel is performed. In contrast, the transient type can be managed conservatively in the absence of any abdominal symptoms suggestive of complicated intussusception. There are no sufficient data in literature to draw any standard therapy or follow up for this rare entity of bowel intussusception.

## Conclusion

Transient intussusception is an extremely rare and challenging condition. The diagnosis can be enigmatic because of the non-specific symptoms and the absence of any pathognomonic clinical features. Imaging techniques, especially CT scan, are helpful in the diagnosis of intussusception. However, laparoscopy offers the advantage of distinguishing transient intussusception from persistent intussusception and other pathological conditions that require immediate surgical treatment.

### Consent

Written informed consent was obtained from the patient for publication of this Case report and any accompanying images. A copy of the written consent is available for review by the Editor of this journal.

## Additional file

Additional file 1:
**Intraoperative video showing multiple segments of small bowel loops observed intussuscepting and reducing spontaneously.**
